# Editorial: New frontiers in safeguarding blood component transfusions: innovative approaches to bacterial risk reduction

**DOI:** 10.3389/fmed.2026.1898289

**Published:** 2026-07-01

**Authors:** Yi Yuan Zhou, Magali J. Fontaine, Ljiljana V. Vasovic, Robert W. Maitta

**Affiliations:** 1 University Hospitals Cleveland Medical Center, Cleveland, OH, United States; 2 Case Western Reserve University School of Medicine, Cleveland, OH, United States; 3 University of Pittsburgh Medical Center, Pittsburgh, PA, United States; 4 University of Pittsburgh School of Medicine, Pittsburgh, PA, United States; 5 Westchester Medical Center Health Network, Valhalla, NY, United States; 6 New York Medical College, Valhalla, NY, United States

**Keywords:** transfusion safety, risk reduction, blood components, blood stewardship, evidence-based, blood supply, pathogen reduction

Blood transfusion is one of the most frequently used medical interventions in modern healthcare, without which many treatments, including surgery, would be impossible. Unlike other medical products, blood supply is supported by voluntary blood donations, which have been on a steady decline due to an aging donor population as well as reduced interest in donation despite increased outreach efforts, both of which were exacerbated by the COVID-19 pandemic (1). To mitigate these supply challenges and ensure sufficient inventory in the future, a dual strategy is being developed by optimizing utilization of blood components using evidence-based guidelines and by advancing methods to extend components shelf-life.

Blood stewardship emphasizes the judicious and evidence-based use of blood components. The need for robust blood stewardship is illustrated in the study by Velasco et al in this special issue, which looked at transfusion appropriateness through a multicenter approach of perioperative transfusion practices in 43 Spanish hospitals (Tamayo-Velasco et al.). Transfusion appropriateness was evaluated based on patient's hemoglobin (Hgb) and clinical conditions using the restrictive transfusion threshold of < 7g/dL with higher thresholds for patients with existing comorbidities. They found that the overall perioperative transfusion rate was about 9.7% half of which constituted transfusion to patients undergoing cardiac surgery, with a smaller but significant percentage represented vascular and orthopedic surgeries. Higher transfusion rates were associated with older patients, presence of comorbidities, lower perioperative Hgb, higher American Society of Anesthesiology physical status (ASA score), and worse postsurgical outcomes. Interestingly, despite having an overall perioperative transfusion rate lower than the international figure, about 43% of transfusions in the study were found to be inappropriate. The authors found that advanced patient age was the factor most strongly associated with inappropriate transfusion. These data highlight an opportunity for potential practice biases that could be remedied by implementation of decision support tools. This represents the first study of its kind from Spain.

Despite the substantial proportion of inappropriate transfusion, the overall decreased rate of perioperative transfusions was attributed to the presence of a patient blood management (PBM) system. A PBM system supports blood stewardship by providing a patient-centered and evidence-based approach to improve a patient's outcome by managing and preserving their own blood so that less blood would be needed perioperatively. To this end, PBM seeks to optimize pre-operative blood cell mass, minimize intra-operative blood loss, and properly manage signs of anemia in the post-operative phase. PBM is explored in the paper by Zhu et al in this special issue, which studied the perioperative management of Factor VII (FVII) deficiency (Zhu et al.). FVII is a vitamin K-dependent protein belonging to the extrinsic coagulation pathway that activates almost immediately in response to tissue damage. Perioperative bleeding is a major concern in patients with FVII deficiency which has heterogeneous presentation. Consequently, there is neither clear consistent correlation between bleeding symptoms and FVII levels, nor clear guidelines in FVII replacement in the perioperative setting. In this study, the authors conducted a retrospective review of the perioperative management of FVII deficiency in 20 patients at a single institution. The authors were unable to find a significant correlation between perioperative hemostasis complications and FVII activity levels. Conversely, the authors noted that replacement of FVII along with anti-fibrinolytic treatment should be considered in the management of severe FVII deficiency especially if there is a history of bleeding. By optimizing FVII levels in the perioperative settings, the authors argue that the risk of hemorrhage can be reduced, thus abrogating the need for extraneous blood component transfusion.

Addressing the complementary challenge of the blood supply shortage, there have been studies into processes that can extend the shelf-life of blood components. Because of their biological nature, bacterial contamination during storage is a major limitation of shelf-life and is especially important in platelet storage, which is normally stored at room temperature. Traditionally, platelet units have a shelf-life of 5 days, 2 of which are reserved for bacterial testing, meaning that at best, blood banks and transfusion services have only 3 days in which to use these units; alternatively, platelet units can have a shelf-life of 7 days if they undergo cultures using large-volume-delayed sampling. With the advent of pathogen-reduction technologies (PRT) there is improved safety of the blood supply. Common PRT technologies include INTERCEPT Blood System (Cerus) and MIRASOL-Pathogen Reduction technology (Terumo BCT). INTERCEPT uses a psoralen compound which selectively binds to nucleic acids and forms strong cross-linkages after UV light exposure, preventing nucleic acid replication of pathogens. By contrast, MIRASOL is based on riboflavin that intercalates with DNA and RNA and causes single strand breaks following UV light exposure. Both technologies inactivate most pathogen contamination and enhance safety of platelets during storage. Some of the challenges of PRT include being implicated in platelet refractoriness and alloimmunization, platelet responsiveness to agonists, and possible diminished platelet viability. These challenges contribute to storage lesions, which are a series of negative structural and functional alterations during storage. The study by Barham et al in this issue seeks to explore the ultrastructural effect of PRT-treated platelets under electron microscopy (Barham et al.). Resting platelets have high mitochondrial activity to meet the adenosine triphosphate (ATP) necessary to maintain cellular processes, structural integrity as well as buffering intracellular calcium, a critical process for platelet activation and aggregation. This study found mitochondrial volume shrinkage and canalicular swelling that begins within 24 hours after collection of the platelet. These morphological changes remained constant for up to 7 days with or without PRT treatment, demonstrating that INTERCEPT and MIRASOL did not contribute to storage lesion of platelet units.

Finally, both arms of blood supply shortage mitigation are addressed in the article by Almadhaani et al that discusses critically important molecules involved in the normal physiological process of red blood cells (RBC) including cellular adherence, formation of extracellular vesicles, maturation, senescence while discussing in detail those factors in storage affecting RBC function (Almadhaani et al.). Thus, this article lays the groundwork for future studies to improve utilization of blood, in particular RBCs while providing a deeper understanding of the mechanism of RBC storage lesions to improve blood supply shelf-life.

In conclusion, this special issue provides insight into strategies for mitigating blood component shortages and improve blood transfusion appropriateness, issues of critical importance in transfusion medicine. Collectively, these contributions highlight the complex challenges facing the blood supply and their broad impact across clinical practice, with a shared focus on improving patient outcomes and advancing transfusion care. However, there are still unmet needs that should be addressed such as improved window period of bacterial detection since low titer or slow growing bacteria could be missed. Similarly, improve current systems and develop new ones that continue to enhance blood component viability and efficacy. Finally, address how to best detect gram negative bacteria which could still represent a challenge for collection facilities in other parts of the world. Therefore, research should continue to improve blood component safety and remain hypervigilant to meet developing challenges facing blood inventories.
